# Cavitary pulmonary metastasis from renal pelvic urothelial carcinoma with pathological evidence of bronchiolar obstruction supporting a check-valve mechanism: a case report

**DOI:** 10.1016/j.rmcr.2026.102431

**Published:** 2026-05-14

**Authors:** Kei Nakano, Tomoki Nakagawa, Haruka Kishi, Shota Fujino, Takashi Ishihara, Masaya Ohara, Kazuhiro Matsuo, Tomoki Higeta, Kie Maita, Hirohito Kobayashi, Masatoshi Yamada, Ryota Masuda

**Affiliations:** a Department of General Thoracic Surgery, Ebina General Hospital, Ebina, Japan; b Department of General Thoracic Surgery, Tokai University School of Medicine, Isehara, Japan; c Department of Urology, Ebina General Hospital, Ebina, Japan; d Department of Pathology, Ebina General Hospital, Ebina, Japan

**Keywords:** Metastatic lung tumor, Renal pelvic cancer, Cavity formation, Check-valve mechanism, Case report

## Abstract

Approximately 4% of metastatic lung tumors exhibit cavitation. Cavitary pulmonary metastases arising from urothelial carcinoma are rare. Renal pelvic cancer accounts for only 3%–5% of all urothelial carcinomas; cavitary pulmonary metastasis from renal pelvic cancer is rare. This report describes pulmonary metastasis from renal pelvic cancer with cavitation. A man in his 50s underwent laparoscopic left nephroureterectomy with hilar lymph node dissection for left renal pelvic cancer 2 years earlier. During follow-up, chest computed tomography (CT) revealed a gradually enlarging lesion with internal cavitation in the right lower lobe. Chest CT demonstrated a 17-mm cavitary nodule in segment 7 of the right lung, enlarged from 4 mm over 1 year. Cavitation was evident even when the lesion was small; tumor diameter and cavity size also increased. The cavity wall remained thin, measuring 1.5 mm in most areas, up to 4 mm, without progressive thickening. Given the rarity of cavitary metastasis from renal pelvic cancer, a surgical biopsy was performed. Thoracoscopic wedge resection revealed a nodular lesion with a grayish-white cavity. Immunohistochemical staining demonstrated GATA3 positivity, confirming pulmonary metastasis. Microscopically, the tumor proliferated while destroying the lung architecture, with small tumor cell clusters within the alveolar spaces. Tumor extension into a bronchiole lacking cartilage resulted in partial luminal obstruction. Here, radiological progression and pathological findings supported cavity formation via a check-valve mechanism. This case provides radiological and pathological evidence supporting a check-valve mechanism of cavitation in pulmonary metastasis and contributes to the differential diagnosis of thin-walled cavitary pulmonary nodules.

## Highlights

• This case provides novel insight into the pathogenesis of cavitary pulmonary lesions through pathological evidence of bronchiolar obstruction.
• A check-valve mechanism due to partial bronchiolar obstruction contributed to cavity formation.
• Clear pathological evidence of bronchiolar obstruction has not been previously demonstrated in cavitary pulmonary metastasis.
• This case aids in the differential diagnosis of thin-walled cavitary pulmonary nodules.

## 1 Introduction

Cavity formation reportedly occurs in 4% of metastatic lung tumors. Squamous cell carcinoma and adenocarcinoma are the most frequent histological subtypes associated with cavitation, whereas pulmonary metastases originating from urothelial carcinomas rarely exhibit this feature. Renal pelvic carcinoma accounts for 3%–5% of all urothelial carcinomas, and cavitary pulmonary metastasis arising from this site is extremely rare. Here, we report a rare case of cavitary pulmonary metastasis from renal pelvic urothelial carcinoma and present radiological and pathological findings suggesting a check-valve mechanism of cavity formation. We present the following case in accordance with the CARE reporting checklist.

## 2 Case presentation

### 2.1 Chief complaint and history

A man in his 50s was referred to our hospital for evaluation of an enlarging cavitary lesion with an associated nodule. The patient reported no hemoptysis or bloody sputum.

Two years prior, the patient underwent a total left laparoscopic nephroureterectomy with hilar lymph node dissection for left renal pelvic cancer in the Department of Urology. On follow-up imaging, computed tomography (CT) revealed an enlarging cavitary lesion in the right lower lobe. The patient was subsequently referred to our department for further assessment.

### 2.2 Past medical history

The patient had left renal pelvic cancer (pT1N0M0, stage I). He had no history of duodenal ulcer, hyperuricemia, hyperlipidemia, or rheumatic disease. The patient had no history of smoking.

### 2.3 Laboratory testing

Sputum analysis for acid-fast bacteria was negative. Further laboratory evaluation revealed the following values: red blood cell count, 423 × 10^4^/μL; white blood cell count, 5260/μL; platelet count, 27.1 × 10^4^/μL; C-reactive protein, 0.02 mg/dL; D-dimer, 0.5 μg/mL; *Cryptococcus* antigen, 0.05 pg/mL (−); *Aspergillus* antigen, 2.6 (+); β-D-glucan, <4.0; T-Spot tuberculosis (−); *Mycobacterium avium complex* antibody (−); carcinoembryonic antigen, 0.7 ng/mL; neuron-specific enolase, 10.9 ng/mL; squamous cell carcinoma antigen, 1.3 ng/mL; cytokeratin 19 fragment, 1.73 ng/mL; and pro-gastrin-releasing peptide, 59.5 pg/mL.

### 2.4 Imaging findings

**Fig. 1:**
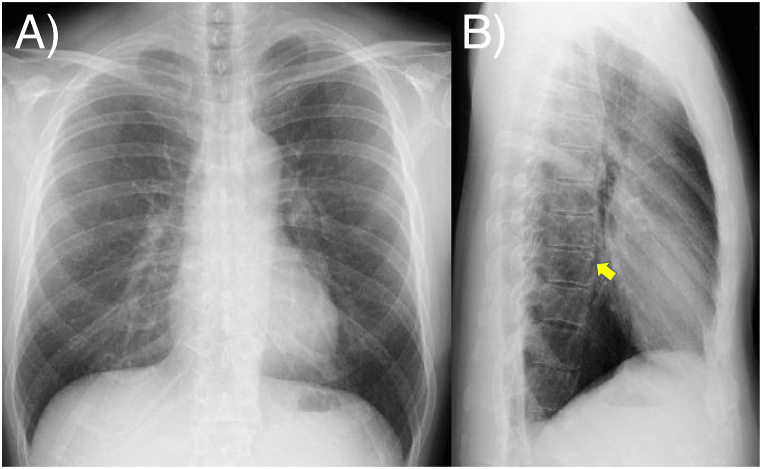
Chest radiograph A) Frontal view: no obvious nodular shadow B) Lateral view: a cavitary nodule overlapping the vertebral bodies in the lower lung field (arrow).

**Fig. 2:**
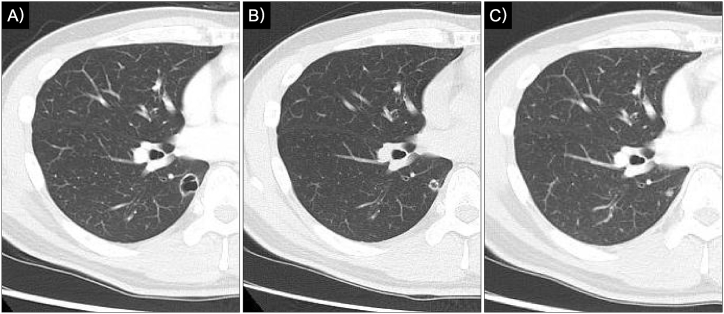
Chest CT findings A) At the time of examination: a cavitary lesion containing a 17-mm nodule. B) Cavity formation became apparent when the tumor measured approximately 7 mm. C) One year prior to evaluation at our department: a 4-mm nodule was present, with no evident cavity formation.

On plain chest radiography, no distinct nodule was apparent on the frontal view (Fig. 1A). On the lateral view, a cavitary nodule overlapping the vertebral bodies in the lower lung field was visible (Fig. 1B, yellow arrow). Chest CT revealed a cavitary nodule in the medial-basal segment (Segment 7) of the right lower lobe, with a maximum diameter of 17 mm. The cavity wall measured approximately 1.5 mm along most of its circumference, with focal thickening of up to 4 mm (Fig. 2A). The lesion had been detectable 1 year earlier, measuring 13 mm in diameter, with an initial cavity approximately 7 mm in diameter (Fig. 2B and C). Over time, the cavity enlarged proportionally with tumor growth, whereas the wall thickness remained unchanged (Fig. 2A–C).

### 2.5 Treatment course

Following a total left laparoscopic nephroureterectomy, renal pelvic cancer recurred in the bladder. Local treatments, including transurethral resection of the bladder tumor and Bacillus Calmette-Guérin instillation, were administered. No distant metastasis requiring systemic chemotherapy was detected, and the overall clinical course was satisfactory. Although pulmonary metastasis from renal pelvic cancer was suspected, cavitary lesions are uncommon in this context. Therefore, a tissue biopsy was planned. Although bronchoscopy and CT-guided biopsy were considered, definitive local diagnosis was deemed challenging. Consequently, thoracoscopic resection of the right lower lobe was performed.

### 2.6 Histopathological findings

**Fig. 3:**
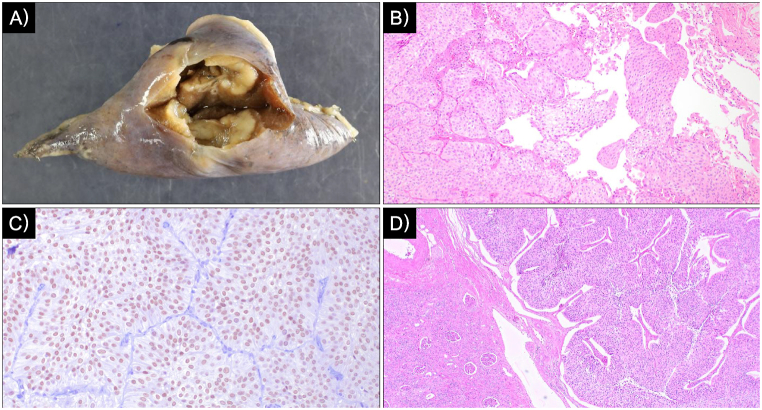
Histopathological findings A) Macroscopic image showing an off-white cavitary lesion with a nodular component B) Tumor cells with irregular macronuclei proliferating in papillary and alveolar patterns, destroying the pre-existing lung architecture. Floating tumor cell clusters are present within alveolar spaces (hematoxylin–eosin staining, objective ×4). C) Immunohistochemical staining demonstrating GATA3 positivity (objective ×20). D) Histology of the primary renal pelvic tumor showing similar features (hematoxylin–eosin staining, objective ×4).

**Fig. 4:**
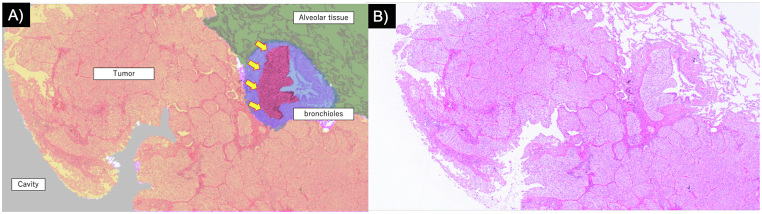
Partial obstruction of cartilage-free bronchioles within the tumor A) Schematic illustration of the histopathological features. Tumor cells within the bronchiolar lumen are highlighted (yellow arrows), demonstrating partial luminal obstruction and the proposed check-valve mechanism. B) Histopathological image showing tumor cells extending into and partially obstructing the lumen of cartilage-free bronchioles within the lesion (hematoxylin–eosin staining, objective ×4).

**Fig. 5:**
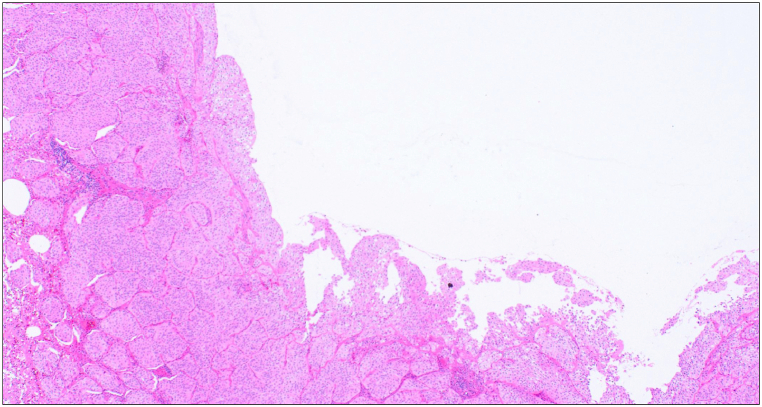
Cavity wall surface Non-necrotic tumor tissue is directly exposed on the cavity surface without a fibrous lining or necrotic debris (hematoxylin–eosin staining, objective ×4).

Macroscopic examination revealed an off-white cavitary lesion with an associated nodule (Fig. 3A). Microscopic analysis demonstrated tumor cells with irregular macronuclei proliferating in papillary and alveolar patterns, disrupting the normal lung architecture (Fig. 3B). Immunohistochemical staining was positive for GATA3 (Fig. 3C), and the histological features were consistent with the primary left renal pelvic cancer (Fig. 3D); hence, pulmonary metastasis from urothelial carcinoma was diagnosed. Tumor cells extended into the lumina of intratumoral cartilage-free bronchioles, causing partial luminal obstruction (Fig. 4). The cavity wall surface lacked significant fibrous tissue or granulation, with a non-necrotic tumor directly exposed on the cavity surface (Fig. 5).

### 2.7 Post-surgical course

Following surgery, systemic chemotherapy for the primary left renal pelvic cancer was planned at the Department of Urology. Treatment is currently ongoing at the time of submission.

## 3 Discussion and conclusions

The differential diagnosis of cavitary pulmonary lesions includes pulmonary tuberculosis, nontuberculous mycobacterial infection, granulomatosis with polyangiitis (Wegener's), pulmonary mycosis, pulmonary suppuration, and primary lung cancer [1]. Among metastatic lung tumors, cavitation has been reported in approximately 4% of patients [2], with squamous cell carcinoma accounting for 69% and adenocarcinoma accounting for the majority of the remainder [3]. Pulmonary metastasis from urothelial carcinoma typically presents as a solid nodule, and cavitary pulmonary metastasis from urothelial carcinoma is rare [4]. As 90%–95% of urothelial carcinomas arise from bladder cancer [5], most reported patients with cavitary pulmonary metastasis from urothelial carcinoma also have primary bladder cancer [[6], [7], [8], [9]]. Nonetheless, upper tract urothelial carcinoma represents only 5%–10% of all urothelial carcinomas, with renal pelvic cancer accounting for approximately 3%–5% [5]. Consequently, cavitary pulmonary metastasis originating from renal pelvic cancer, as observed in the present patient, is exceedingly rare.

Two primary mechanisms have been proposed to explain cavity formation in metastatic lung tumors: 1) intratumoral necrosis (resulting from tumor growth with central ischemia and chemotherapy-induced necrosis) and 2) a check-valve mechanism, in which the tumor partially obstructs peripheral airways [1,10]. In necrosis-related cavitation, central liquefactive necrosis is considered the dominant process. The resulting cavity walls are typically irregular and relatively thick, and pathological examination often reveals necrotic tissue accompanied by inflammatory cell infiltration [11]. Conversely, in cavity formation via the check-valve mechanism, partial tumor obstruction in cartilage-free bronchioles may promote airway collapse and restrict expiratory airflow, creating a functional check-valve. Subsequent air trapping and increased intraluminal pressure may drive cavity formation or enlargement [12,13].

This mechanism is not unique to metastatic lung tumors and has been implicated in multiple pathologies. Peripheral airway stenosis associated with bronchiolitis, pneumonia, smoking, and other conditions contributes to cyst and bulla formation [[13], [14], [15]] and has been linked to giant bullae [16] and progressive emphysema [17]. Similar mechanisms have been described in the formation of pneumatoceles secondary to airway stenosis following infections and traumatic injuries [18]. In both the central and peripheral airways, structural fragility due to insufficient bronchial cartilage or cartilage defects can produce a check-valve effect, as observed in hereditary conditions such as congenital lobar emphysema [19,20]. Tumor lesions, including lepidic lung adenocarcinomas, may similarly cause partial airway obstruction and contribute to cavitation [12]. In malignant tumors, cavity formation is generally associated with wall thickening [21], whereas benign lesions more commonly exhibit thin walls (≤4 mm) [1,13]. Nevertheless, relatively thin walls have also been reported in malignant tumors when cavitation arises from the check-valve mechanism [12,22].

In the present patient, systemic chemotherapy was not administered, excluding treatment-related necrotic cavitation. Serial imaging revealed that the cavity developed while the tumor remained relatively small, and it enlarged in parallel with tumor growth. Notably, most of the cavity wall remained thin (approximately 1.5 mm, with a maximum of approximately 4 mm), with no apparent increase in wall thickness relative to tumor size. Pathological examination showed no evidence of necrotic tissue or inflammatory cell infiltration, findings inconsistent with necrosis-driven cavitation. These findings argue against necrosis-related cavitation and instead support cavity formation via a check-valve mechanism due to partial bronchiolar obstruction.

Pathology demonstrated tumor infiltration into the alveoli, with numerous microtumor clusters partially obstructing the bronchioles connected to the cavity. Given that metastatic lung tumors are typically established via a hematogenous route [10,23], the tumor likely extended into adjacent bronchioles and alveoli following implantation in the lung parenchyma. This suggests that partial obstruction of cartilage-free bronchioles generated a check-valve mechanism, with air trapping and increased intratumoral pressure contributing to cavity formation and progressive enlargement. Additionally, the absence of fibrous lining and direct exposure of non-necrotic tumor tissue were consistent with airflow into the cavity. To our knowledge, clear pathological images demonstrating partial bronchiolar obstruction associated with cavitary pulmonary metastasis have not been clearly demonstrated in previous case reports.

Cavitary pulmonary metastasis from upper tract urothelial carcinoma, particularly renal pelvic cancer, is uncommon, and imaging-based differential diagnosis may be challenging when the primary disease has followed a favorable course. Additionally, significant wall thickening may be absent even in malignant cavitary lesions formed via a check-valve mechanism, as observed in this patient. Therefore, in patients with similar imaging findings, histological diagnosis is essential. When bronchoscopy and CT-guided biopsy are not feasible, surgical approaches, including thoracoscopic lung biopsy, should be considered.

Although rare, renal pelvic carcinoma can present with cavitary pulmonary metastasis, in which a check-valve mechanism may play a contributory role. When cavitary pulmonary lesions are identified during the clinical course of renal pelvic carcinoma, pulmonary metastasis should be included in the differential diagnosis.

## CRediT authorship contribution statement

**Kei Nakano:** Writing – review & editing, Writing – original draft, Conceptualization. **Tomoki Nakagawa:** Visualization, Validation, Supervision, Methodology. **Haruka Kishi:** Visualization. **Shota Fujino:** Visualization. **Takashi Ishihara:** Visualization. **Masaya Ohara:** Visualization. **Kazuhiro Matsuo:** Visualization. **Tomoki Higeta:** Visualization. **Kie Maita:** Visualization. **Hirohito Kobayashi:** Validation, Supervision, Resources, Project administration. **Masatoshi Yamada:** Validation, Supervision, Resources, Project administration. **Ryota Masuda:** Visualization, Validation, Supervision, Resources, Project administration, Conceptualization.

## Consent for publication

Written informed consent to publish this case report and accompanying images was obtained from the patient and their family.

## Data statement

All data generated or analyzed during this study are included in this article. Further information is available from the corresponding author upon reasonable request.

## Funding

This research did not receive any specific grant from funding agencies in the public, commercial, or not-for-profit sectors.

## Declaration of competing interest

The authors declare no competing interests.
