# Supplementary material for: Cavitary pulmonary metastasis from renal pelvic urothelial carcinoma with pathological evidence of bronchiolar obstruction supporting a check-valve mechanism: a case report
Source: Respir Med Case Rep. 2026 May 14;62:102431. doi: 10.1016/j.rmcr.2026.102431 (PMC13197699; doi:10.1016/j.rmcr.2026.102431)
Supplement: Multimedia component 1 [file mmc1.pdf]

CARE case report guidelines

CARE Checklist of information to include when writing a case report

(cc) BY-NC-ND

Topic	Item	Checklist item description	Reported on Line
Title	1	The diagnosis or intervention of primary focus followed by the words "case report"	Page1 line1-3
Key Words	2	2 to 5 key words that identify diagnoses or interventions in this case report, including "case report"	Page2 line30-31
Abstract (no references)	3a	Introduction: What is unique about this case and what does it add to the scientific literature?	Page1 line6-10
	3b	Main symptoms and/or important clinical findings	Page1 line10-12
	3c	The main diagnoses, therapeutic interventions, and outcomes	Page1-2 line17-24
	3d	Conclusion—What is the main "take-away" lesson(s) from this case?	Page2 line25-27
Introduction	4	One or two paragraphs summarizing why this case is unique (**may include references**)	page3 line34-39
Patient Information	5a	De-identified patient specific information.	page3-4 line44-58
	5b	Primary concerns and symptoms of the patient.	page3-4 line44-58
	5c	Medical, family, and psycho-social history including relevant genetic information	page3-4 line44-58
	5d	Relevant past interventions with outcomes	page3-4 line44-58
Clinical Findings	6	Describe significant physical examination (PE) and important clinical findings.	N/A
Timeline	7	Historical and current information from this episode of care organized as a timeline	page3-4 line44-58
Diagnostic Assessment	8a	Diagnostic testing (such as PE, laboratory testing, imaging, surveys).	Page4-5 line60-80
	8b	Diagnostic challenges (such as access to testing, financial, or cultural)	Page5-6 line82-91
	8c	Diagnosis (including other diagnoses considered)	Page6 line 93-103
	8d	Prognosis (such as staging in oncology) where applicable	Page7 line 105-108
Therapeutic Intervention	9a	Types of therapeutic intervention (such as pharmacologic, surgical, preventive, self-care)	Page7 line 105-108
	9b	Administration of therapeutic intervention (such as dosage, strength, duration)	N/A
	9c	Changes in therapeutic intervention (with rationale)	N/A
Follow-up and Outcomes	10a	Clinician and patient-assessed outcomes (if available)	N/A
	10b	Important follow-up diagnostic and other test results	Page7 line 105-108
	10c	Intervention adherence and tolerability (How was this assessed?)	Page7 line 105-108
	10d	Adverse and unanticipated events	Page7 line 105-108
Discussion	11a	A scientific discussion of the strengths AND limitations associated with this case report	Page7-8 line 111-124
	11b	Discussion of the relevant medical literature **with references**.	Page8-9 line125-149
	11c	The scientific rationale for any conclusions (including assessment of possible causes)	Page11 line179-184
	11d	The primary "take-away" lessons of this case report (without references) in a one paragraph conclusion	Page11 line179-184
Patient Perspective	12	The patient should share their perspective in one to two paragraphs on the treatment(s) they received	title page page2 line22-24
Informed Consent	13	Did the patient give informed consent? Please provide if requested	Yes ☑ No ☐
